# Microalgae: towards human health from urban areas to space missions

**DOI:** 10.3389/fpls.2024.1419157

**Published:** 2024-08-16

**Authors:** Xiulan Xie, Abdul Jaleel, Jiasui Zhan, Maozhi Ren

**Affiliations:** ^1^ Laboratory of Space Biology, Institute of Urban Agriculture, Chinese Academy of Agricultural Sciences, Chengdu, China; ^2^ Zhengzhou Research Base, State Key Laboratory of Cotton Biology, School of Agricultural Sciences, Zhengzhou University, Zhengzhou, China; ^3^ Department of Integrative Agriculture, College of Agriculture and Veterinary Medicine, United Arab Emirates University, Al Ain, United Arab Emirates; ^4^ Department of Forest Mycology and Plant Pathology, Swedish University of Agricultural Sciences, Uppsala, Sweden

**Keywords:** interstellar migration, microalgae, natural products, synthetic biology, extreme space environment

## Abstract

Space exploration and interstellar migration are important strategies for long-term human survival. However, extreme environmental conditions, such as space radiation and microgravity, can cause adverse effects, including DNA damage, cerebrovascular disease, osteoporosis, and muscle atrophy, which would require prophylactic and remedial treatment *en route*. Production of oral drugs *in situ* is therefore critical for interstellar travel and can be achieved through industrial production utilizing microalgae, which offers high production efficiency, edibility, resource minimization, adaptability, stress tolerance, and genetic manipulation ease. Synthetic biological techniques using microalgae as a chassis offer several advantages in producing natural products, including availability of biosynthetic precursors, potential for synthesizing natural metabolites, superior quality and efficiency, environmental protection, and sustainable development. This article explores the advantages of bioproduction from microalgal chassis using synthetic biological techniques, suitability of microalgal bioreactor-based cell factories for producing value-added natural metabolites, and prospects and applications of microalgae in interstellar travel.

## 1 Introduction

Institutes and space agencies operated by several countries have proposed or implemented successful manned space exploration projects. For example, the China Manned Space Agency is planning a manned moon landing in 2030, whereas the United States proposed sending 1 million people to Mars to establish a human settlement by 2050, attracting global attention (National Geographic, 2016). Space tourism and interstellar migration have garnered interest as essential research fields in the continuous advancement of manned commercial spaceflight technology. Therefore, effects of space travel on the long-term survival of humans are of interest to researchers.

Although many research institutes, companies, and independent groups worldwide have formulated strategic interstellar travel plans, the harmful biological effects of the extreme space environment pose an obstacle to successfully implementing these strategies (Liu et al., 2021). Space radiation and microgravity induce excessive free radical production, resulting in DNA damage, mitochondrial dysfunction, osteoporosis, muscle atrophy, cerebrovascular disease, and central nervous system damage (Afshinnekoo et al., 2020; da Silveira et al., 2020). Moreover, health issues, such as cerebrovascular disease, cancer, fatigue, and depression, are also prevalent in urban environments on Earth and are linked to air pollution, climate change, and lifestyle habits (Khraishah et al., 2022). During space travel, in addition to physical protection, appropriate exercise, timely psychological intervention, and the availability of nutrients, natural metabolites, and drugs are crucial in preventing and treating diseases. Health-related substances, especially drugs that can mitigate or eliminate deleterious health effects, are considered essential for successfully executing manned space travel. However, delivering these substances from Earth to distant locations across space is expensive and unsustainable (Aunon-Chancellor et al., 2020). Thus, *in situ* production of oral drugs is a crucial factor in long-duration space exploration missions and interstellar migration.

Plants produce numerous natural secondary metabolites, and their production has been improved via natural selection, artificial domestication, and intelligent cultivation (Tian et al., 2021). Plant secondary metabolites are vital in alleviating suboptimal health and disease. Plant-based eating regimens include bioactive substances, such as phytochemicals, that promote health and prevent disease (Mullins and Arjmandi, 2021). Phytoactive ingredients exert positive physiological effects on the body, such as improving immunity, alleviating cardiovascular disease, regulating blood sugar levels, reducing blood lipid levels, and promoting digestion (Khalid et al., 2022). These effects are vital for mitigating or eliminating damage to the human body caused by the extreme environmental conditions in space.

**Figure 1:**
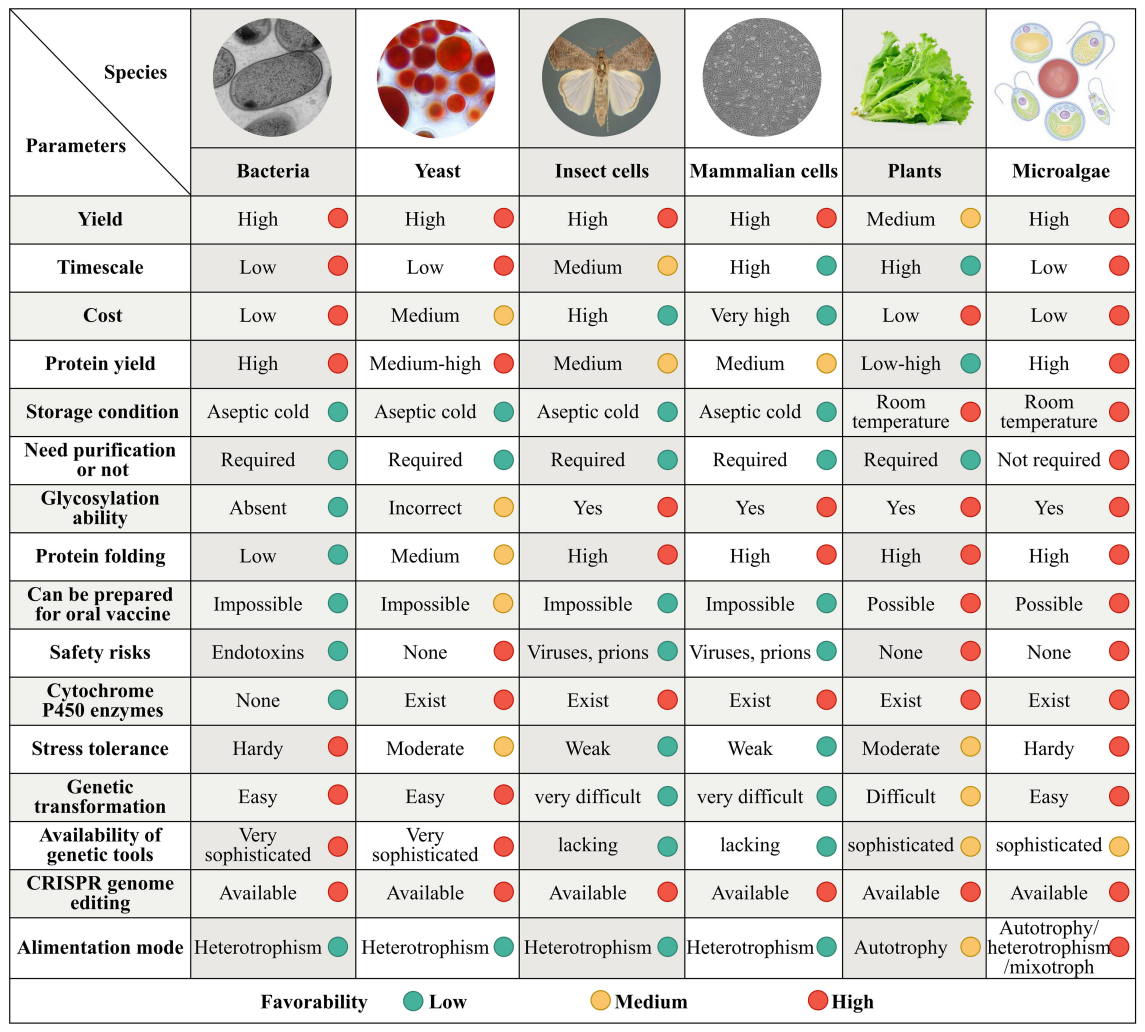
Comparison of the microalgal chassis to those of other organisms for the synthesis of medical metabolites. Synthetic biology expression systems have been established in bacteria, yeast, insect and mammalian cell lines, plants, and microalgae. Although the production parameters vary substantially among these systems, the microalgal system outperforms the others in terms of yield, timescale, quality, production cycle, cost, safety, high by-product concentrations tolerance, key precursors in cells abundance, enzyme expression of heterologous pathways conditions, and the gene-modification tools availability. Each parameter is divided into low, medium, and high favorability represented by green, yellow, and red circles, respectively.

Further, among the approximately 8.7 million species of living organisms on Earth, microalgae are one of the most promising candidates for addressing these pressing issues owing to their unique biological, nutritional, and functional characteristics, as well as their great efficiency as a resource (Torres-Tiji et al., 2020) (**Figure 1**). Moreover, microalgae have the advantages of high photosynthetic efficiency, fast growth rate, low resource usage, high energy efficiency, edibility, high production efficiency, rich and comprehensive nutritional profile, convenient genetic enhancement, and large-scale production. More importantly, microalgae have a wide and rapid adaptability. Microalgae are one of the earliest photosynthetic life forms on Earth, and their persistence in extreme environments during all past mass extinction events demonstrates high stress tolerance, strong resistance, and adaptability (Benton, 1995), making them a highly sustainable option in the context of extreme environmental conditions in space.

From this perspective, we have considered the health issues faced by human beings in space and propose an overall strategy for preventing and responding to these health issues. We have also considered the suitability of microalgae for meeting health needs during space travel, given their unique biological and genetic properties. Additionally, we propose technological and scientific advances that may enable high-efficiency industrial production of microalgae in the resource-limited environment of space.

## 2 Natural metabolites show potential for supporting health during space travel

Radiation and microgravity are critical environmental factors affecting human health during long-duration space flight and exploration (Blue et al., 2019). Gravity on Mars is only one-third that on Earth, and the radiation dose on the surface of Mars is five times that inside the International Space Station orbiting Earth. Humans would be exposed to ionizing radiation of up to 1000 millisieverts in the Martian environment (Nangle et al., 2020). Ionizing radiation induces excessive production of free radicals, leading to oxidative stress. Additionally, the average temperature on Mars is −80°F, and extremely low temperatures can also induce oxidative stress (Munguira et al., 2020).

**Figure 2:**
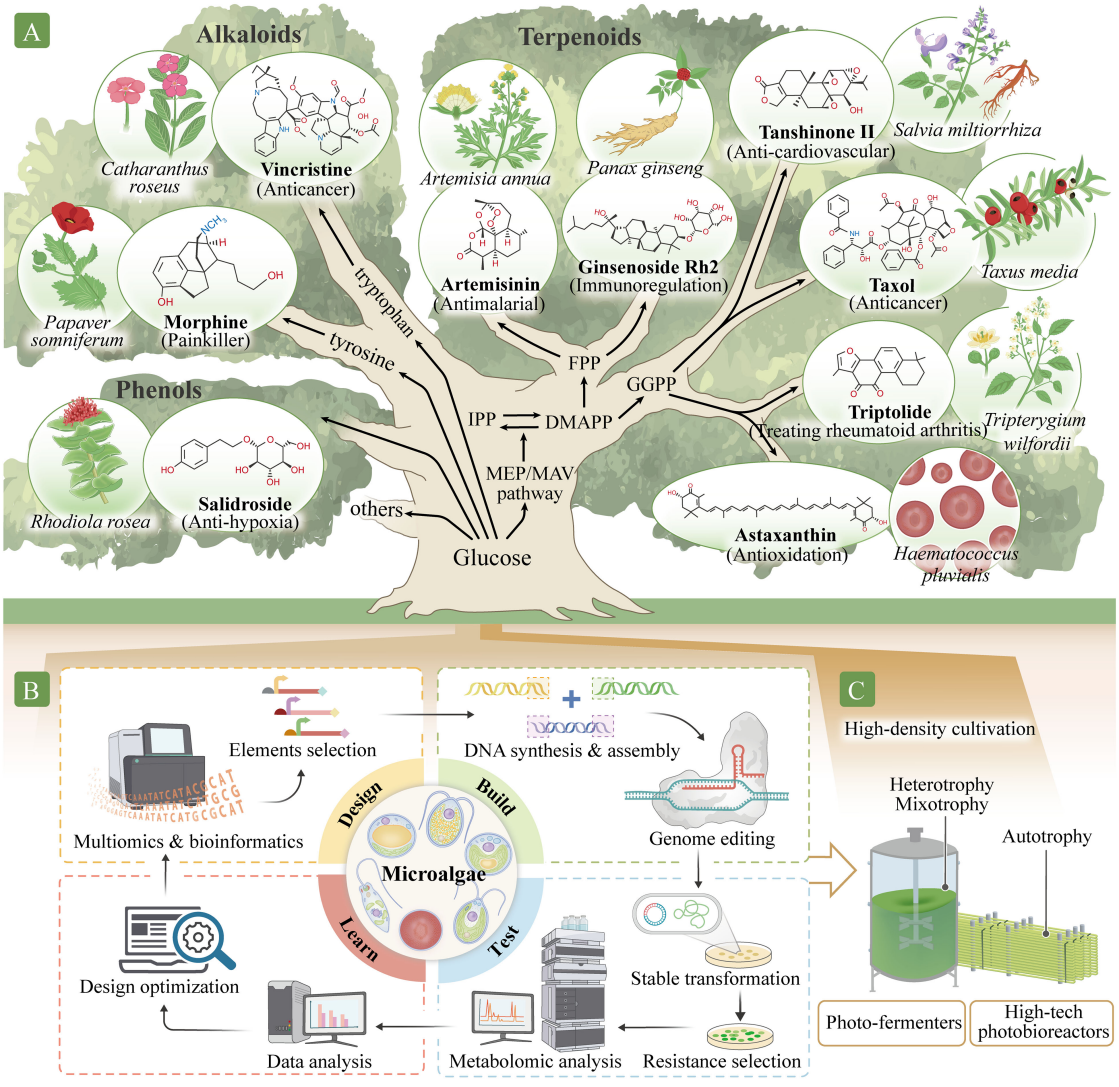
Production of high-value natural metabolites using a microalgal cell factory. **(A)** Identifying pathways to synthesize high-value secondary metabolites in the microalgal chassis. These high-value secondary metabolites include phenols, such as salidroside; alkaloids, such as morphine and vincristine; and terpenoids, such as artemisinin, paclitaxel, triptolide, tanshinone, ginsenoside, and astaxanthin. Isopentenyl pyrophosphate (IPP) and dimethylallyl pyrophosphate (DMAPP) are C5 precursors for all classes of terpenoids. These molecules also participate in the synthesis of farnesyl pyrophosphate (FPP), C15, artemisinin precursors, ginsenoside, and geranylgeranyl pyrophosphate (GGPP), C20, as well as tanshinone, paclitaxel, triptolide, astaxanthin, and other carotenoids precursors. **(B)** Establishing synthetic biology systems for metabolite production. Establishment involves four phases: Design, Build, Test, and Learn. In the Design phase, the target components and their metabolite pathways are identified and designed using multi-omics and bioinformatics methods. In the Build phase, DNA is synthesized, assembled, and modified. In the Test stage, genetic transformation into the microalgal chassis is performed, and positively transformed algae are identified and analyzed. In the Learn phase, data from the Test phase are analyzed and used to predict or optimize the next cycle. **(C)** Application for large-scale microalgal production modes. The microalgal systems can be improved with new production approaches, such as combining photobioreactor and fermentation technology for industrial production.

Abundant antioxidants in plants, such as astaxanthin, lutein, fucoxanthin, ginsenosides, salidroside, polyphenols, anthocyanins, and lycopene, scavenge or competitively bind to free radicals, thereby preventing biological damage caused by excessive exposure to ionizing radiation and improving cellular response (Rahaman et al., 2023). Astaxanthin, found mainly in *Haematococcus pluvialis*, is a tetraterpenoid ketone carotenoid that is one of the most potent natural antioxidants (**Figure 2A**). Its antioxidant activity is considerably higher than that of tea polyphenols, anthocyanins, glutathione, coenzyme Q10, vitamin C, and vitamin E. Astaxanthin effectively eliminates various types of free radicals in cells, improves cell regeneration, maintains homeostasis, and reduces senescent cells accumulation (Alugoju et al., 2022). Ionizing radiation also causes DNA damage; insufficient DNA repair may lead to cell death, mutations, chromosome rearrangements, and even carcinogenesis. Paclitaxel is a tetracyclic diterpenoid compound extracted from *Taxus*, one of the best-known anticancer and antitumor drugs (Jiang et al., 2024) (**Figure 2A**). Vincristine (Zhang et al., 2022b; Gao, 2023), ginsenoside Rg3 (Xia et al., 2022), and dihydroartemisinin (Dai et al., 2021) also exhibit antitumor effects (**Figure 2A**).

Microgravity can lead to space syndromes, such as psychological dysfunction, head fluid accumulation, cardiovascular and cerebrovascular system dysfunction, bone loss, and muscle atrophy. Natural compounds, such as ginsenosides in *Panax ginseng*, salidroside in *Rhodiola rosea*, tanshinone in *Salvia miltiorrhiza*, and astaxanthin in *H. pluvialis* exert substantial cardio–cerebrovascular protective effects (Alugoju et al., 2022; Li et al., 2022a). Ginsenosides (such as Rb1, Rb2, Rb3, Rc, Rd, Rg3, Rh2, and PD) exhibit multiple pharmacological activities, including immune system regulation, enhancing cardiovascular health, and improving myocardial ischemia, in addition to their protective effects against fatigue, tumors, and depression (Li et al., 2022a) (**Figure 2A**). Salidroside is an active ingredient extracted from the rhizome of *R. rosea* that exerts many pharmacological effects, including anti-fatigue and anti-hypoxia effects, immune regulation, and free radical scavenging (Zhang et al., 2021) (**Figure 2A**). Tanshinones, such as tanshinone I, tanshinone IIA, tanshinone IIB, dihydrotanshinone I, and cryptotanshinone, are fat-soluble phenanthrenequinone compounds extracted from the rhizome of *S. miltiorrhiza* that have extensive cardio–cerebrovascular protective effects (Lu, 2021; Ma et al., 2021) (**Figure 2A**). For example, sodium tanshinone IIA sulfonate is widely used to treat coronary heart disease. Artemisinin derived from *Artemisia annua* is effective for malaria treatment (Zhang et al., 2022a) and has other pharmacological effects, such as antitumor, immune regulation, antibacterial, and anti-cardiovascular disease effects. Triptolide from *Tripterygium wilfordii* is used to treat rheumatoid arthritis (Tong et al., 2021), and morphine from *Papaver somniferum* is an opioid analgesic (Guo et al., 2018) (**Figure 2A**).

However, plant metabolite yields are limited owing to their low production levels, long accumulation periods, complex extraction processes, and unsatisfactory quality (Liu et al., 2022). Furthermore, many plant species cannot grow or show limited potential for growth on other planets (Liu et al., 2021), restricting the application of their secondary metabolites for medical applications during space travel and migration. Therefore, exploring new synthetic biological chassis and developing technologies for the mass production of plant secondary metabolites is essential to fulfill the medical needs associated with space travel and migration.

## 3 Selection of suitable synthetic biological chassis hinges for mass production

Genes encoding numerous active ingredients and their synthetic pathways in products such as terpenoids, alkaloids, and phenolic compounds have been identified (Li and Gaquerel, 2021). These developments have provided the necessary foundational knowledge for industrial production of high-value-added metabolites for human consumption. Synthetic biological expression systems have been constructed in bacteria, yeast, insect cell lines, mammalian cell lines, and plants. These expression platforms have different advantages and limitations in terms of yield, quality, production cycle, cost, safety, tolerance to high product concentrations, abundance of key precursors in cells, conditions for enzyme expression of heterologous pathways, and availability of gene modification tools (**Figure 1**).

Traditional microbial chassis in bacteria (such as *Escherichia coli*, *Bacillus subtilis*, and *Corynebacterium glutamicum*) and yeast (such as *Saccharomyces cerevisiae* and *Pichia pastoris*) have been widely studied and are used for heterologous protein expression, as these organisms are fast growing, easy to genetically manipulate, and have high productivity. However, using bacterial hosts often results in protein misfolding and aggregation into insoluble inclusion bodies in recombinant expression. These hosts also lack the membrane compartments required to effectively express membrane-bound enzymes such as cytochrome P450 monooxygenase, which limits their production of natural products. Their products may also be contaminated with bacterial endotoxins that restrict their long-term development as chassis (Russo et al., 2019).

Although yeast has endomembrane compartments that are more suitable for expressing eukaryotic products, it grows as a single cell. It can also only be cultivated at scale through heterotrophic metabolism, heavily relying on costly organic carbon sources such as glucose and sucrose, leading to high production costs (Srinivasan and Smolke, 2020). Although recombinant proteins and vaccines produced in insects and mammalian cells (such as CHO and HEK293) can undergo post-translational modifications (Courdavault and Papon, 2022), yeast remains the main chassis used in licensed pharmaceutical protein production (Zhao et al., 2021). However, this chassis type has limited biosynthetic capacity, high production costs, and a high risk of pathogen contamination, severely restricting its application in the large-scale production of secondary metabolites.

Plant chassis, such as tobacco (Molina-Hidalgo et al., 2021), rice (Zhu et al., 2022), and duckweed (Lam and Michael, 2022) have clear advantages in phototropism, membrane protein expression, precursor supply, product tolerance, and compartmentalization compared with model microbial chassis that are heterotrophic (such as *E. coli* and yeast) and animal cell lines. Additionally, plants have mechanisms for post-translational modifications, are not susceptible to zoonotic pathogens, and have potential as production platforms. In recent years, there have been breakthroughs in technologies required to utilize plants to produce therapeutic proteins and vaccines (Lin et al., 2023). However, industrial production using plants is limited by their long growth cycles and challenges associated with genetic transformation.

Microalgae is the generic name used for a class of photosynthetic unicellular microorganisms, including *Arthrospira*, *Chlorella*, *Chlamydomonas*, *Dunaliella*, *Nostoc*, *Aphanizomenon*, and *Haematococcus*. These organisms are photosynthetic cell factories with characteristics similar to those of microorganisms, animals, and plants, and live in a variety of habitats. Microalgae production systems have high biosynthetic precursors, flexible production modes, and high scalability. They are highly efficient at carbon fixation, are environmentally friendly, and have high levels of biological safety (**Figure 1**). Consequently, they offer a possible solution for mass production of secondary metabolites.

## 4 Microalgae potential for metabolite mass production

Microalgae are one of the earliest photosynthetic organisms on Earth, and have survived five mass extinctions to date. They are among the most promising candidates for ensuring human health and safety in space owing to their unique biological, nutritional, and functional characteristics (Jahn et al., 2023). Microalgae are superior to other metabolite production systems, such as bacteria, yeast, insect and mammalian cell lines, and higher plants in terms of yield, quality, cost efficiency, environmental adaptability, biochemical composition, and technological requirements for biochemical enrichment and industrial production (**Figure 1**).

### 4.1 Biosynthesis challenges despite biosynthetic precursors availability

Microalgae contain cellular compartments and internal membrane systems, such as chloroplasts, that provide ideal environment for different metabolites synthesis (Jiao et al., 2020). In eukaryotic algae, cytosolic mevalonate and plastidic 2-C-methyl-d-erythritol 4-phosphate (MEP) pathways generate isopentenyl pyrophosphate (IPP, C5) and dimethylallyl pyrophosphate (DMAPP, C5) (Wichmann et al., 2020). Genomic predictions indicate the presence of different prenyltransferase classes in microalgae that allow synthesis of farnesyl pyrophosphate (FPP, C15) (precursor of artemisinin, ginsenoside, and other dolichols) in the cytosol and geranylgeranyl pyrophosphate (GGPP, C20) (precursor of tanshinone, paclitaxel, triptolide, astaxanthin, and other carotenoids) in the chloroplast (**Figure 2A**). Given their shared evolutionary history with higher plants, microalgae may possess innate capacities for the heterologous production of plant terpenoids by producing terpene synthases (Athanasakoglou and Kampranis, 2019). However, the *de novo* biosynthesis of high-value secondary metabolites in algal systems remains challenging despite the development of several modular cloning toolkits (Crozet et al., 2018). This is likely owing to distinct capacity to adapt to the metabolic pull of further metabolic engineering for heterologous isoprenoid production.

### 4.2 Various microalgae show potential for natural metabolites synthesis

Microalgae synthesize natural pigments, such as chlorophylls, phycobilins, and carotenoids. Among these, tetraterpenoids (C40) including astaxanthin, lutein, fucoxanthin, canthaxanthin, zeaxanthin, β-carotene, and β-cryptoxanthin exhibit multiple biological activities beneficial to human health, such as antioxidant, anti-angiogenic, anti-cancer, and anti-osteoporotic effects and protective effects on the bones, brain, blood vessels, eyes, liver, and skin (Leong et al., 2022; Patel et al., 2022; Russell et al., 2022). *Dunaliella salina* has been used for commercial production of β-carotene, which accounts for 10% of its biomass on a dry weight basis (Luo et al., 2021). *Haematococcus pluvialis* is one of the richest natural sources of astaxanthin, which is a red pigment of the carotenoid class known as a “super-antioxidant” (Mullins and Arjmandi, 2021). *Isochrysis zhangjiangensis* can provide high fucoxanthin productivity of 3.06 mg/L/d by balancing high biomass concentration and fucoxanthin content (Li et al., 2022b).

The number and activity of cytochrome P450 enzymes (CYPs) directly affect metabolite profiles. High number of CYPs is typically found in microalgae than in animals or yeast, accounting for up to 0.2% of metabolite-coding genes in some species (Hansen et al., 2021). For example, *C. reinhardtii* has 39 CYP genes, with discovery of additional microalgal CYPs likely to continue (Hansen et al., 2021). Microalgae also have readily available common enzyme cofactors and metabolic precursors. Microalgal chassis are suitable for the synthesis and post-translational modification of secondary metabolites, as their genes are similar to those of plant sources of candidate genes, resulting in metabolites with high enzyme activity and yield.

### 4.3 Environmental factors affecting microalgae production and metabolite synthesis

Microalgae have long been proposed for use in space life support systems to recover CO_2_ and provide food and medicine directly or indirectly to astronauts (Zhang et al., 2020; Mapstone et al., 2022). On Earth, microalgae survive in many harsh environments, including extreme temperatures, high salinity, osmotic pressure, and ultraviolet radiation (Cvetkovska et al., 2022; Montuori et al., 2023). Plant secondary metabolites, mainly phenols, terpenoids, and alkaloids, play an important role in plant response to environmental stress, such as reducing oxidative damage by acting as antioxidants (Sun and Fernie, 2024). Many microalgae have been reported to live in extreme cold environments, such as *H. pluvialis*, *Desmodesmus* sp., *C. vulgaris*, and *Chlamydomonas priscuii* (Leon-Vaz et al., 2023). While certain microalgal cells can form resting spores/cysts, many polar algae appear to remain in a vegetative state during winter. Fahrion et al. (2021) provided an overview of space experiments conducted in low Earth orbit regions with photobioreactors over the last 30 years. The experiments highlighted that gas-liquid transfer phenomena are different under microgravity conditions, which inevitably can affect the cultivation process and oxygen production. Helisch et al. (2020) reported a stable heterogeneous long-term cultivation of *Chlorella vulgaris* SAG 211-12 in a novel microgravity-capable membrane raceway photobioreactor for 188 days. Based on the closed loop system, a successive biomass growth of up to a maximum of 12.2 g/L with a maximum global volumetric productivity of 1.3 g/L/d was achieved (Helisch et al., 2020). Some microalgae, such as *Limnospira indica*, have a strong resistance to radiation (survives at least 6400 Gy of gamma rays), and, thus, will not be negatively affected by an increase in ionizing radiation in space (Badri et al., 2015). In addition to microgravity, radiation, hypoxia, and extreme temperature, low pressure is also prevalent on other planets, and even a vacuum exists in space. Growth rate of *C. reinhardtii* CC-1690 cells decreased, carbon dioxide absorption rate increased, and oxygen production rate remained unchanged under lower pressure (Wagner and Posten, 2017). It can be seen that microalgae have a strong adaptability to various environments, and moderate extreme environments can promote synthesis of secondary metabolites. However, experimental demonstration to prove that a microalgal-based system could fulfill the requirements for a space-based bioregenerative life support system under comparable spaceflight power, mass, and environmental constraints must be conducted.

## 5 Microalgal pathways require improvement for use in space travel

As mentioned above, many natural metabolic pathways and precursors present in higher plants are also found in microalgae. Additionally, microalgae have advantages in terms of membrane protein expression, product tolerance, compartment synthesis, abundant CYPs presence, and related activity (**Figure 1**). However, known microalgae have limited metabolite types and synthesis capabilities. For example, fewer alkaloids exist in microalgae than in terrestrial plants. Additionally, no terpene synthase genes were detected in green algae from 158 transcriptomes (47 charophytes and 111 chlorophytes) and 31 genomes (Jia et al., 2022). Nevertheless, prospects for the large-scale production of many high-value products from microalgae using synthetic biology, gene editing, and metabolic engineering techniques appear promising (**Figure 2B**). Several strategies may be required to improve the production of microalgal products for space travel and migration.

### 5.1 Designing molecular switches for inducible expression systems to enhance efficiency

Although plant secondary metabolites enhance the environmental adaptability of plants, their accumulation at high levels can cause cell toxicity. Cellular tolerance to the heterologous expression of secondary metabolites is an important and common challenge in synthetic biology. Inducible gene expression (Ochoa-Fernandez et al., 2020) and genome editing systems (Wang et al., 2020) can achieve specific spatiotemporal expression and gene knockouts, respectively. These approaches allow metabolic processes to be spatiotemporally regulated to a certain extent and are reversible, thus providing a solution to the trade-off solution between the efficient synthesis of metabolites and cell growth. Although our understanding of some light-signaling pathways (red and far-red light) (Han et al., 2019) and hormone-signaling pathways during the evolution of microalgae is limited (Lu and Xu, 2015), the natural light- and hormone-induced promoters of higher plants can be utilized to develop synthetic molecular switches for inducible expression systems in microalgae. In addition, the reported inducible promoters, such as salt-inducible *CrGPDH3* promoter from *C. reinhardtii* (Beltran-Aguilar et al., 2019), alcohol-inducible AlcR-P_alcA_ system from *Aspergillus nidulans* (Lee et al., 2018), would promote the synergistic and efficient expression of secondary metabolites in microalgae while reducing the interference of endogenous elements.

### 5.2 Strengthening the precursor supply to ensure efficient production

Primary metabolism, such as that of glucose and amino acids, provides essential precursor pools to generate plant secondary metabolites (**Figure 2A**). Increasing the precursor supply ensures that sufficient source compounds levels are directed to the pathways of interest for natural product biosynthesis, thus eliminating any possibility of the depletion of synthetic building blocks. For example, IPP and DMAPP are precursors for terpenoids. Various high-value terpene metabolites (**Figure 2A**) are produced by the action of different enzymes, and precursor synthesis is promoted by overexpressing genes encoding the rate-limiting enzymes of the MEP pathway, such as 1-deoxy-D-xylose-5-phosphate synthase and GGPP synthase (Li et al., 2019). Zhao et al. (2022) introduced the isopentenol utilization pathway into *C. reinhardtii*, and further introduced diphosphate isomerase and limonene synthase, and the highest limonene production reached 117 µg/L.

### 5.3 Importance of overexpression of key enzymes and inhibition of competitive enzymes

Rate-limiting enzymes are crucial in determining metabolic flux. Overexpression of an enzyme with an inducible promoter (heterologous) enhances complete conversion of a substrate into products (or intermediate products) in a pathway. For example, overexpression of native *ORANGE* with a strong light-inducible promoter in *C. reinhardtii* enhances the accumulation of several carotenoids, including β-carotene, α-carotene, lutein, and violaxanthin (Yazdani et al., 2021). In contrast, CRISPR/CRISPR-associated nuclease 9 (CRISPR-Cas9), RNA interference (RNAi), and other antisense techniques can be used to inhibit the expression of enzymes related to the synthesis of “dead-end” intermediates and end-product analogs (by-products) (Liu et al., 2023). For example, phytoene is a colorless carotenoid that accumulates in *D. salina* V-101 when the expression of the phytoene desaturase gene is downregulated via RNAi and antisense technology (Srinivasan et al., 2017). Song et al. (2020) generated a double knockout mutant (lycopene epsilon cyclase and zeaxanthin epoxidase) by the CRISPR-Cas9 ribonucleoprotein-mediated knock-in system, resulting in a 60% higher zeaxanthin yield (5.24 mg L^− 1^) and content (7.28 mg g^− 1^) than that of the parental line.

### 5.4 Introducing transporters and other expression elements to improve efficiency

Notably, ATP-binding cassette transporters promote secretion of metabolites synthesized by microalgal cells, thereby inhibiting negative feedback effects of metabolites on cells and reducing cytotoxicity. For example, the ATP-binding cassette family transporter, ABCG2, is a potential taxane transporter candidate that can be mediated by expelling paclitaxel from cells (Yu et al., 2023). Astaxanthin binding protein, a novel family of carotenoprotein, was found to bind to astaxanthin in microalgae and is useful for providing valuable astaxanthin in a water-soluble form (Yao et al., 2023). Meanwhile, skillfully applying of self-cleaving 2A peptides (Plucinak et al., 2015), fusion proteins, and gene clusters can help reduce the size of vector fragments (Zhan et al., 2022; Liu et al., 2023). Technological changes, such as subcellular localization, key site mutagenesis, codon optimization, and synthetic intron diffusion, can help improve the efficiency of product synthesis.

## 6 Advantages of industrial microalgal production for use in space travel

Most microalgae can thrive under light and dark conditions and be cultivated under photo-autotrophic, heterotrophic, and mixotrophic environments (**Figure 2C**). In the absence of light, microalgae use dissolved organic compounds as energy and carbon sources; meanwhile, light and carbon dioxide are used as energy and carbon sources, respectively, for photosynthesis (Godrijan et al., 2022).

Given limited availability of resources in outer space, microalgal production on other planets will require greater efficiency than production on Earth, and strategies for efficient waste recycling, such as water and carbon dioxide, should be established. For example, photobioreactor and fermentation production systems can be powered by photovoltaics, eliminating the need for other fuel sources (**Figure 2C**). Furthermore, microalgae can absorb carbon dioxide produced by astronauts, recycle small amounts of wastewater, and produce oxygen, food, and medicinal metabolites to partially support food and healthcare needs of the astronauts. Some microalgae, such as *Arthrospira maxima*, *A. platensis*, *H. pluvialis*, *C. pyrenoidosa, D. salina*, *C. reinhardtii*, *Nannochloropsis gaditana*, and *Euglena gracilis*, have already been used in food, commercial, and technology applications, with substantial advances in their large-scale production. Using these biological techniques to create high-quality edible microalgae species cannot only produce drugs on Earth before space missions, but more importantly can use compact bioreactors during space missions to produce directly edible drugs. The health issues related to interstellar travel and migration could be effectively addressed using these microalgal chassis to directly produce high-value secondary metabolites.

## 7 Necessity for ongoing research

With continued exploration of outer space, space travel and interstellar migration have emerged as vital options for supporting human survival in the future. The role of natural plant metabolites in human healthcare is expected to increase, and the functional characterization and efficient production of plant metabolites are expected to emerge as important research areas owing to their important role in human resistance to extreme environmental conditions, such as space radiation and microgravity. Microalgae show potential for supporting the mass production of secondary metabolites for space travel and migration, given their rapid growth properties, metabolite richness, strong adaptability, and ease of genetic modification. Quality, concentration, and diversity of medicinal metabolites in microalgae can be genetically engineered on existing chassis using advanced biotechnologies, such as gene editing and synthetic biology, and microalgae can be cultured at high densities in photo-fermenter production systems. As we mentioned in the biotechnological modification strategy, it is recommended that possible drug production adopts induced expression and culture in a closed culture system. Chassis cells grow rapidly when the metabolite synthesis gene is not expressed under normal culture conditions. When cell growth reaches stability, the induction method can be applied, and the metabolite synthesis gene will be highly induced and expressed, which not only avoids the inhibitory toxicity of the metabolites to the plant cells during the growth process but also greatly improves the synthesis speed and purification efficiency of the metabolites by inducing the synergistic high-efficiency expression. This microalgae-based production model also has the potential to contribute to general medicine and the health of humans on Earth. It can be applied to daily life, as well as marine reefs, plateau deserts, desert gobi, land and sea border defense, deep-sea deep space and emergency response. This perspective provides a foundation for future research on the human health and sustainable extraplanetary expansion of human civilization.

## Data Availability

The original contributions presented in the study are included in the article/supplementary material. Further inquiries can be directed to the corresponding authors.
